# Pharmacological interventions targeting the gut–brain axis in neurological disorders: mechanisms and translational applications

**DOI:** 10.3389/fnins.2026.1806532

**Published:** 2026-04-01

**Authors:** Xu Li, Wenke Zhou, Shuran Yang, Xiangkai Huang, Kuihong Zheng

**Affiliations:** 1 Department of Radiology, The Sixth Medical Center of PLA General Hospital, Beijing, China; 2 Department of Neurosurgery, Xiangya Hospital, Central South University, Changsha, Hunan, China; 3 Xiangya School of Medicine, Central South University, Changsha, Hunan, China; 4 School of Traditional Chinese Medicine BUCM, Beijing University of Chinese Medicine, Beijing, China

**Keywords:** gut–brain axis, interventions, neurodegenerative diseases, pharmacological, precision medicine, therapies

## Abstract

The microbiota–gut–brain axis represents a complex bidirectional communication network linking the gastrointestinal system and the central nervous system and has been increasingly recognized as a key contributor to neurological and psychiatric disorders. Growing evidence indicates that alterations in gut microbiota composition and function can influence brain development and function through neural, immune, endocrine, and metabolic pathways, thereby modulating neuroinflammation, neurotransmission, and blood–brain barrier integrity. Dysregulation of this axis has been implicated in a range of conditions, including Parkinson’s disease, Alzheimer’s disease, multiple sclerosis, autism spectrum disorder, depression, anxiety, and stroke. Recent pharmacological advances have identified the microbiota–gut–brain axis as a promising therapeutic target. Current strategies focus on modulating shared pathophysiological mechanisms rather than disease-specific endpoints and include microbiota-directed interventions, immune–inflammatory modulators, neurotransmitter-targeting agents, and approaches aimed at restoring intestinal and blood–brain barrier function. In this review, we summarize the core mechanisms underlying microbiota–gut–brain axis dysfunction and organize existing pharmacological strategies according to their primary targets. By integrating evidence across multiple disorders, we provide a mechanism-oriented framework to support future drug development and precision therapeutic approaches for brain disorders.

## 1 Introduction

Alzheimer’s disease and associated dementias (ADRD), as well as Parkinson’s disease (PD), are the most particularly challenging neurodegenerative diseases globally whose prevalence and burden of disease keep increasing recently reaching about 60 million individuals each year, and the loss of healthy life years persists (Anwar et al., 2024). Interestingly, of all the neurological conditions, PD is the one with the most age-standardized increase in incidence rates, whereas ADRD has the most significant effect on the rise in disability-adjusted life years (DALYs), a fundamentally crucial metric of the burden of disease (Anwar et al., 2024; Huang et al., 2023; Bashir et al., 2025).

The diagnostic issue of neurodegenerative conditions (including AD and PD) is gaining momentum, increasing its global proportion, and this proportion is strongly developing paradoxically in its growth relationship with the socioeconomic progress. Upon achieving the upper-middle-income level, the disease burden does not only fail to drop, but it is also increasing; despite regional health inequalities continue to expand. This contradiction is the effect of the combination of various modernization factors such as aging of the population, profound changes in the lifestyle, and the grow of social pressure (Ji et al., 2024; Xiaopeng et al., 2025). The multidimensional demand of treatment is largely unmet in relation to the multidimensional requirement of treatment as the contrasting extreme to this excessive disease burden. Available drugs, including levodopa, treat the signs and symptoms mainly. Despite the promising effects of recently approved monoclonal antibodies, including Lecanemab, on disease modification, their clinical benefits with low efficacy and safety concerns have caused a lot of contention (Bashir et al., 2025; Mahase, 2024; Auclair-Ouellet et al., 2025). The treatment is very unequal, being costly and slow diagnosis, which compels most patients to miss the golden period. At the same time, the failure rates and costs of the traditional mode of drug development have led to a rediscovery in the innovative methods used in isometric drug development such as drug repurposing (Auclair-Ouellet et al., 2025).

To conclude, neurodegenerative diseases already face the dual threat of the high burden and the lack of therapeutic choices (Xiaopeng et al., 2025). The bidirectional communication system, which has been referred to as the gut–brain axis (GBA), has proven to be the new frontier in combating this problem with the intervention strategies having the potential to explain the origins of peripheral diseases, to identify early biomarkers, and to establish new disease-modifying therapies (Auclair-Ouellet et al., 2025).

In response to these therapeutic limitations, a diverse array of alternative and adjunctive strategies are being actively explored. Among these, neuromodulation techniques have garnered significant attention. Approaches such as deep brain stimulation (DBS) and transcranial magnetic stimulation (TMS) offer the ability to directly modulate pathological neural circuits, providing symptomatic relief in conditions like PD where pharmacological options become limited or complicated by motor fluctuations (Cleary and Bucholz, 2021; Faraji et al., 2025b). Concurrently, there is a growing recognition that the pathophysiology of neurodegenerative and neuropsychiatric disorders extends beyond the central nervous system (CNS) itself, involving complex interactions with peripheral systems. This has led to a surge of interest in the GBA as a novel therapeutic frontier. Notably, these two seemingly distinct approaches—neuromodulation and GBA-targeting—are not mutually exclusive. Emerging evidence suggests that peripheral interventions, such as vagus nerve stimulation (VNS), can directly engage the GBA, influencing both gut function and central inflammation, thereby bridging the gap between these therapeutic modalities (Faraji et al., 2025b).

**Figure 1:**
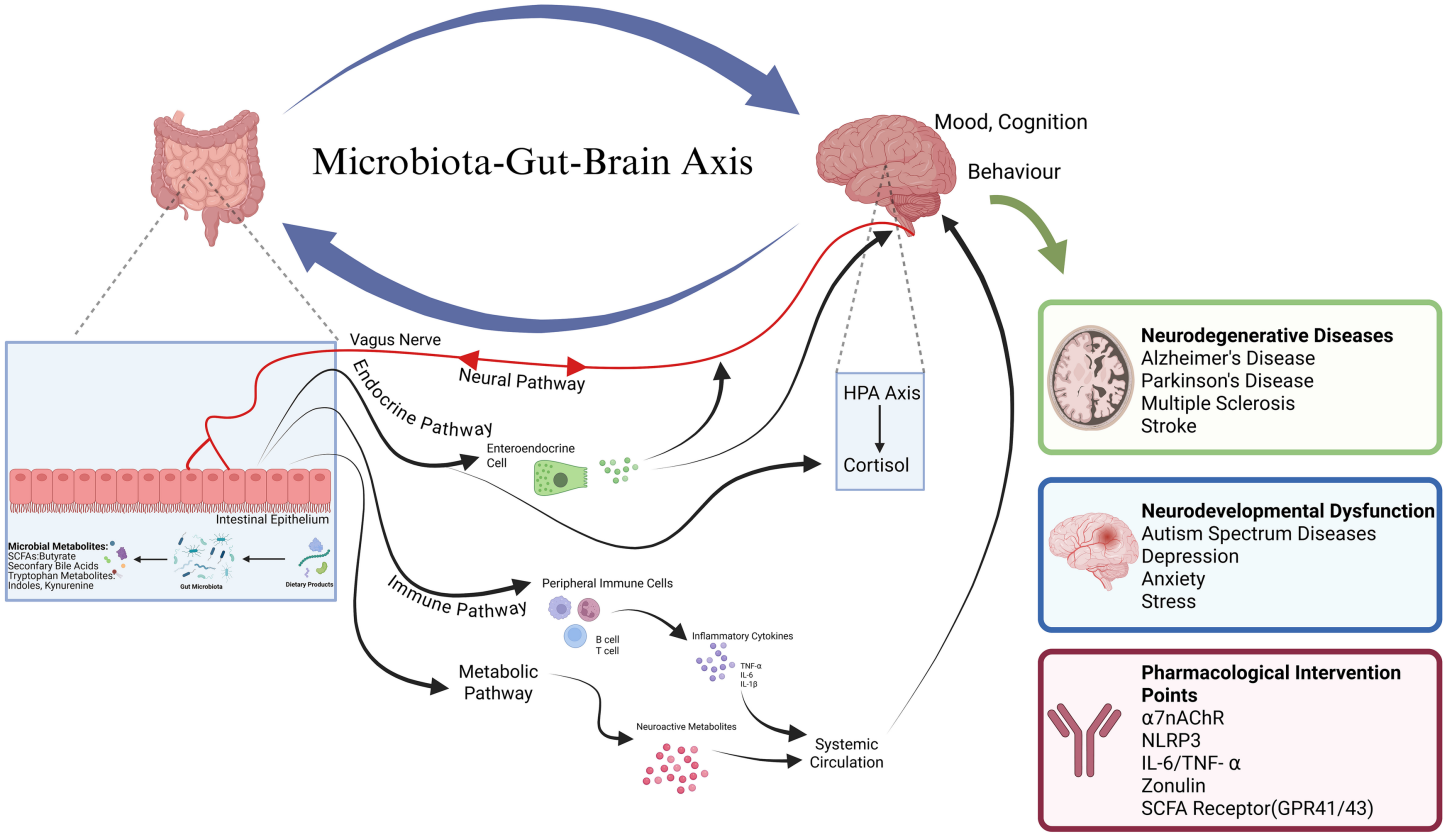
The GBA: mechanisms and pharmacological targets. Schematic representation of the bidirectional communication pathways linking the gut microbiota to the CNS. Left (gut lumen): the gut microbiota produces neuroactive metabolites, including SCFAs, BAs, and tryptophan-derived metabolites, which act locally on enteroendocrine cells, immune cells, and enteric neurons. Middle (gut wall and periphery): signals from the gut are transmitted to the CNS via multiple routes: (1) neural pathway: direct activation of vagal afferent fibers by microbial metabolites or enterochromaffin cell-derived serotonin; (2) endocrine pathway: gut hormones and microbial metabolites enter the circulation and act on distant organs or cross the BBB (BBB); (3) immune pathway: microbial components (e.g., LPS) and metabolites modulate peripheral immune cells, altering cytokine profiles that influence CNS function; (4) metabolic pathway: circulating SCFAs and other metabolites directly modulate neuronal and glial function. Right (CNS): these signals collectively regulate neuroinflammation, neurotransmitter balance, synaptic plasticity, and BBB integrity, thereby influencing mood, cognition, and behavior. Pharmacological intervention points (highlighted in colored boxes) include: VNS, SCFA receptors (GPR41/43), cytokine signaling pathways (TNF-α, IL-6, NLRP3 inflammasome), and tight junction modulators targeting the intestinal barrier and BBB. Dysregulation of these pathways contributes to the pathophysiology of PD, AD, MS, autism spectrum disorder, depression, and stroke.

According to the latest years, the GBA—also referred to as the microbiota-MGBA (MGBA) to emphasize the role of the gut microbiome has become one of the key ideas to consider the pathogenesis of neurological conditions and new treatment options (Loh et al., 2024). This is a two-way network of communication which incorporates a combination of various pathways such as neural, endocrine, immune and metabolic systems (Dong and Mayer, 2024) (Figure 1). The brain controls intestinal motility, secretion and permeability through the autonomic nervous system, and the hypothalamic–pituitary–adrenal axis in the so-called top-down pathway, thus modulating the composition and activity of the gut microbiota (Loh et al., 2024). On the other hand, the bottom-up pathway implies that the microbiota of the gut and its metabolites interact with the CNS using numerous mechanisms. The vagus nerve (VN) serves as a critical bidirectional communication highway, transmitting real-time information about the gut microenvironment to the CNS. Beyond its well-characterized role in conveying satiety and visceral sensory signals, the VN is increasingly recognized as a key modulator of higher-order brain functions. It exerts neuroprotective effects by regulating neuroinflammation, modulating neurotransmitter systems (e.g., norepinephrine, serotonin), and promoting the release of neurotrophic factors (Faraji et al., 2025b). This positions the VN as a central node not only in immune regulation but also in the broader pathophysiology of neurodegenerative and neuropsychiatric disorders. There are also neuroactive compounds dependent on microbes of the gut produced or regulated as gamma-aminobutyric acid (GABA), serotonin, short-chain fatty acids, and bile acids (BAs) are critical endocrine and metabolic signals controlling CNS homeostasis and function (Zhuang et al., 2024). Both intestinal and blood–brain barrier (BBB) permeability are important in supporting homeostasis of this two-way communication. By interfering, their translocation enables harmful substances to be transferred and this is a shared pathological process in a number of neuropsychiatric conditions (Zhuang et al., 2024; Khan et al., 2025). In line with this, the focus on the microbiome to restore the integrity between the MGBA has become a very promising proposed solution in terms of neurodegenerative illnesses such as Alzheimer’s and Parkinson’s (Loh et al., 2024; Warren et al., 2026).

In the paper, a narrative review of pharmacologically-oriented intervention strategies based on the MGBA will be provided. It will cover the mechanisms of action of probiotics, prebiotics, and synbiotics; approaches targeting key microbial metabolites; as well as dietary and lifestyle modifications. Additionally, the paper will evaluate the current evidence supporting their use in specific neurological disorders and discuss the existing challenges (Auclair-Ouellet et al., 2025).

In this review, we adopt a broad and mechanism-centric view of ‘pharmacological interventions.’ While acknowledging that classical pharmacology primarily deals with defined chemical compounds (small molecules or biologics) with specific molecular targets, we also recognize that interventions such as probiotics, prebiotics, and fecal microbiota transplantation (FMT) exert their effects through defined pharmacological principles. They modulate host physiology by altering the concentration of bioactive metabolites (e.g., SCFAs, BAs), engaging host immune receptors (e.g., TLRs), and influencing neurotransmitter signaling. Therefore, we consider these microbiome-targeted or GBA-modulating therapies as part of an expanded pharmacological toolkit, focusing on their mechanism of action rather than their origin as living organisms.

## 2 Key pharmacological targets and pathways of the MGBA

### 2.1 Microbial metabolites

Metabolites of the gut microbiota serve as the most direct pharmacological messengers mediating communication along the MGBA, with short-chain fatty acids (SCFAs) playing a central role, while bile acids and tryptophan derivatives perform complex and critical dual functions. SCFAs, primarily including butyrate, propionate, and acetate, are produced by gut microbial fermentation of dietary fiber (Senarath et al., 2024). They can cross the BBB and exert extensive neuroprotective effects through multiple mechanisms (Senarath et al., 2024). First, SCFAs act as potent histone deacetylase (HDAC) inhibitors, especially butyrate, which promotes the expression of neuroplasticity-related genes via epigenetic regulation (Lee et al., 2024; Castillo-González et al., 2025). Second, they serve as ligands for G protein-coupled receptors (GPCRs) such as GPR41, GPR43, and GPR109A. For example, butyrate activates GPR109A on brain endothelial cells, leading to upregulation of tight junction proteins like occludin and claudin-1, thereby enhancing the integrity of the BBB (Lee et al., 2024). In AD models, SCFAs not only inhibit the formation of neurotoxic amyloid-β (Aβ) oligomers but also modulate excessive neuroinflammation through interactions with glial cells (Senarath et al., 2024). Additionally, SCFAs provide anti-inflammatory and homeostatic regulation of microglia and promote neuroprogenitor cell proliferation via GPR41/43 signaling (Lee et al., 2024).

BAs, as cholesterol derivatives, are increasingly recognized for their roles within the CNS (Loh et al., 2024; Romero-Ramírez and Mey, 2024). They can enter the brain via the circulatory system and may also be synthesized locally within the CNS. BAs exhibit distinct neuroprotective properties. Indeed, chenodeoxycholic acid (CDCA) is reported to decrease the infarct size in stroke models via antagonism of N-methyl-D-aspartate receptors having the GluN2B subunit, thus, lowering glutamate excitotoxicity (Monteiro-Cardoso et al., 2024). Tauroursodeoxycholic acid (TUDCA) is effective in treating pathological phenotypes in a variety of neurodegenerative disease models (Romero-Ramírez and Mey, 2024). Such protective properties can be presumably explained by the fact that BAs stimulate the Takeda G protein-linked receptor 5 (TGR5), a signaling pathway that is effective in inhibiting neuroinflammation (Romero-Ramírez and Mey, 2024). Nevertheless, BAs too have dual functions in that at the pathological processes such as that of liver failure, the excessive levels of BAs can have an effect of altering the lipid bilayer of the BBB, thereby elevating its permeability and making it potentially dangerous to the nervous system (Romero-Ramírez and Mey, 2024).

The first and rate-limiting step of the kynurenine pathway is catalyzed by the enzyme indoleamine 2,3-dioxygenase 1 (IDO1), which converts tryptophan into N-formylkynurenine (Wang et al., 2025b; Kwidzinski and Bechmann, 2007). IDO1 serves as a critical molecular link between systemic inflammation and subsequent neurotoxic metabolite production. Its expression is strongly induced by pro-inflammatory cytokines, particularly interferon-γ (IFN-γ) and tumor necrosis factor-α (TNF-α), which are elevated during peripheral infections, chronic inflammation, or autoimmune conditions (Kwidzinski and Bechmann, 2007; Fujigaki et al., 2006; Haroon et al., 2012). In addition to IFN-γ, lipopolysaccharide (LPS) can also trigger IDO1 expression through IFN-γ-independent mechanisms involving the p38 mitogen-activated protein kinase (MAPK) and nuclear factor-κB (NF-κB) pathways (Fujigaki et al., 2006).

Within the CNS, IDO1 is expressed in microglia and infiltrating macrophages upon exposure to these pro-inflammatory signals (Kwidzinski and Bechmann, 2007; Haroon et al., 2012; Gan and Lee, 2025). Once activated, IDO1 diverts tryptophan metabolism away from serotonin synthesis toward the kynurenine pathway, leading to the production of downstream metabolites with opposing neuroactive properties (Wang et al., 2025b; Gan and Lee, 2025). Thus, IDO1 functions as a “molecular switch” that, when triggered by peripheral or central inflammation, shifts the metabolic balance toward neurotoxic kynurenine metabolites such as quinolinic acid (QUIN), thereby linking systemic immune activation to CNS pathology (Kwidzinski and Bechmann, 2007; Haroon et al., 2012).

Tryptophan metabolites provide central networks within neuroimmunoregulations (Kondo et al., 2024). The major tryptophan metabolism pathway is the kynurene (Kyn) one, the products of which have paradoxical effects on neural health. Kynurenic acid (KYNA) is a neuroprotective NMDA receptor antagonist and QUIN is an NMDA receptor agonist on one hand and on the other hand respectively, that leads to excitotoxicity and neuroinflammation (Romero-Ramírez and Mey, 2024). Kynurenine pathway metabolic imbalances (i.e., metabolic shift toward neurotoxic products such as QUIN) (Kondo et al., 2024). In addition, some tryptophan may be degraded by intestinal microbes to indole and indole derivatives (e.g., indole-3-propionic acid). Also, some of the tryptophan is converted by gut microbiota into indole and indole derivatives (e.g., indole-3-propionic acid). These toxins stimulate the aryl hydrocarbon receptor (AhR), a protein that helps to sustain the integrity of the intestinal barrier and immune homeostasis, and thus indirectly affects brain health (Yin et al., 2021).

### 2.2 Neuroimmunoregulation

The gut microbiota engages the microglia-immune cell of the brain in systematic, dynamic, and lifelong control, a specialized bi-directional communication pathway known as the microbiota-microglia axis. This regulation plays a significant role in the initiation, progression, and resolution of inflammation in the CNS and forms a central part of neuroimmunoregulation (Song et al., 2025). AD, PD, and multiple sclerosis (MS) are excellent examples of these behavioral disorders with dysregulation of this axis as a major causative factor.

The gut microbiota and its metabolites directly shape the phenotype and function of microglia through circulatory, neural, and other pathways. Core molecular mechanisms include: SCFAs, such as butyrate, which act as HDAC inhibitors (Song et al., 2025) and activate GPCRs (e.g., GPR43). These activities not only promote glial cell polarization toward the anti-inflammatory M2 phenotype but also provide energy substrates to support their homeostatic functions (Wang et al., 2026). Meanwhile, gut microbiota-derived indole metabolites (e.g., indole-3-lactic acid) activate the AhR, reprogramming microglial metabolism by shifting it toward oxidative phosphorylation, thereby suppressing excessive inflammatory responses and supporting synaptic function (Zhu et al., 2026). Conversely, under dysbiotic conditions, elevated levels of pro-inflammatory substances such as LPSs from Gram-negative bacterial cell walls strongly activate the Toll-like receptor 4 (TLR4)/myeloid differentiation factor 88 (MyD88) pathway on microglia surfaces. This drives polarization toward the pro-inflammatory M1 phenotype, resulting in massive release of TNF-α, and interleukin-1β (IL-1β), triggering chronic neuroinflammation (Chen et al., 2026). Furthermore, specific gut bacteria and their components—such as outer membrane vesicles secreted by *Bacteroides fragilis* and the bacterial protein Amuc_1100—can cross or indirectly influence the BBB. Upon uptake by microglia, these molecules directly inhibit pathological activation by activating pathways like TLR2 (Wang et al., 2026; Chen et al., 2026).

In disease settings, this regulation exhibits pronounced pathological effects. For instance, in AD models, dysbiosis (such as abnormal proliferation of *Bacteroides fragilis*) suppresses peripheral immune cells from producing critical signals like granulocyte-macrophage colony-stimulating factor, thereby impairing microglial phagocytic capacity and reducing their ability to clear Aβplaques (Rooks and Garrett, 2016). In PD, gut-derived α-synuclein pathology may be transmitted via pathways such as the VN, activating brain microglia and amplifying neuroinflammation. Directly, the phenotype and function of microglia are defined by the gut microbiota and its metabolites via the circulatory, neural, and any other pathway. This has also been the cause of research leading to the development of what is known as microglial priming, in which aberrant microbial signaling in early life or chronic changes of the epigenetic and metabolic status of microglia alters the epigenetic state of the latter. This causes a primed state of the baseline thus causing overreacting inflammatory responses when there is an exposure to the secondary insults such as infection or trauma hence predisposing to neurodegenerative diseases (He et al., 2024).

Given the pivotal role of the “microbiota-glia axis,” targeting this pathway is emerging as a highly promising therapeutic strategy. Reshaping the gut microbiota through probiotics, prebiotics, specific microbial metabolites (e.g., SCFAs, indole-3-lactic acid), or engineered microbial carriers can precisely reprogram microglia from “drivers” to “regulators” of neuroinflammation (Wang et al., 2026; Zhu et al., 2026; Chen et al., 2026). Preclinical evidence shows that dietary flavonoid supplementation effectively suppresses microglial activation and improves cognitive function by modulating the microbiota and elevating butyrate levels. This heralds a novel paradigm in precision neuropharmacology: systematically regulating “brain immunity” through gut-targeted interventions.

While selective serotonin reuptake inhibitors (SSRIs) such as fluoxetine are primarily prescribed for enhancing central serotonergic transmission in mood disorders, emerging evidence suggests that their pharmacological actions may also involve significant interaction with the gut–brain axis. A majority of the body’s serotonin is synthesized by enterochromaffin cells in the gut, where local serotonergic signaling influences motility, secretion, and immune function (Sjöstedt et al., 2021). SSRIs not only block serotonin reuptake centrally and peripherally, but have been shown to alter gastrointestinal physiology and microbial composition, potentially via direct antimicrobial activity against commensal taxa (Sjöstedt et al., 2021). These antimicrobial effects have been proposed to arise from mechanisms such as efflux pump inhibition or disruption of microbial transport functions (Ait Chait et al., 2020). In clinical cohorts, differences in gut microbiota composition have been associated with differential SSRI treatment outcomes, implicating microbiome-metabolite interactions in therapeutic response (Jiang et al., 2024). Such pleiotropic pharmacological effects—where a CNS-targeted drug exerts peripheral modulation of gut microbial ecology and related neuroactive metabolites—underscore the importance of considering gut–brain axis dynamics in both the mechanistic understanding and clinical application of SSRIs (Xu et al., 2023).

### 2.3 Neuroendocrine and vagal pathways

As the body’s primary neuroendocrine system for stress response, the HPA axis is finely regulated by the gut microbiota (Ortega et al., 2021). By producing metabolites such as SCFAs and modulating immune responses, the gut microbiota “programs” the development and stress responses of the HPA axis (Ortega et al., 2021). Under normal physiological conditions, gut–brain axis signaling contributes to the homeostatic regulation of the HPA axis, supporting adaptive neuroendocrine stress responses (Cryan et al., 2019). Chronic stress and gut dysbiosis have been associated with dysregulated and prolonged HPA axis activation, potentially resulting in sustained elevations of circulating glucocorticoids, including cortisol. Persistent hypercortisolism is known to exert neurotoxic effects, contributing to neuronal damage and structural atrophy in stress-sensitive brain regions such as the hippocampus, thereby impairing emotional and cognitive function—a pattern widely observed in stress-related neuropsychiatric disorders (Bertollo et al., 2025). Preclinical studies further suggest that gut microbiota-dependent modulation of the HPA axis exhibits circadian variability. Experimental models demonstrate that microbial rhythmicity influences diurnal glucocorticoid secretion and host stress reactivity, indicating that microbiota–host interactions may participate in the temporal organization of neuroendocrine responses rather than exerting static effects (Bertollo et al., 2025; Thaiss et al., 2014). Importantly, much of the evidence linking microbiota to circadian regulation of the HPA axis derives from animal and mechanistic studies, and its quantitative relevance in humans remains under active investigation. This creates new information about the original rhythmic symptoms of the stress related disorders (Tofani et al., 2025). Also, it has been found by research that there are gut-brain communication processes that do not rely on the conventional HPA-cortisol system. As an example, chronic stress may impair the metabolism of vitamin B6 by changing the composition of the intestinal microbiota, which will then cause abnormal behavior in individuals (Qing et al., 2025). This gives the indication that there are several pathways of gut-brain signaling.

Unlike the rather slow humoral regulation through the HPA axis, the VN offers the simplest physical interface and smoothest signal transduction between the gut and the brain (Gwak and Chang, 2021). It’s afferent fibers innervate the gut in great detail, enabling it to pick on a change of all layers of the intestine environment in an acute way (Jameson et al., 2025). Current landmark research offers conclusive findings that certain gut microbiota and metabolites serve as primary signals to activate the VN (Jameson et al., 2025). As an example, microbial metabolites, including SCFAs and BAs are direct agonists of particular receptors on vagal nerve terminals and stimulate particular neuronal groups (Jameson et al., 2025). It is these interactions that turn the chemical signals into neural electrical signals that are relayed back to the brainstem via the solitary tract nucleus to the regions of the brain. One such described pathway includes the select microflora (e.g., *Bacteroides fragilis*) triggering cholinergic cells on the intestinal epithelium. These cells produce acetylcholine that selectively stimulates the VN (Jia et al., 2026). The resultant neural impulses eventually pathway to the hippocampus, causing fast antiepileptic effects and the long term neuroprotective effects. This constitutes a definite functional loop: “certain microbes-intestinal epithelial cells-VN-brain target regions.”

Based on this, the VN can serve as an important information superhighway, transmitting real-time information about the gut microbiota dynamics to the CNS (Wang et al., 2025a). Beyond its role as an afferent information conduit, the VN, and its therapeutic modulation via VNS, exerts pleiotropic effects on brain function that extend well beyond the cholinergic anti-inflammatory pathway discussed above. Accumulating evidence indicates that VNS can directly influence neuronal excitability and plasticity. For instance, VNS has been shown to enhance the release of key neurotransmitters, including norepinephrine and serotonin, which are critical for mood regulation and cognitive function. This mechanism may underlie its therapeutic efficacy in treatment-resistant depression and epilepsy. Furthermore, VNS promotes neuroprotection and neurogenesis by upregulating brain-derived neurotrophic factor (BDNF) and other trophic factors, potentially slowing disease progression in neurodegenerative conditions (Faraji et al., 2025b).

Critically, the interaction between VNS and the gut microbiota represents a novel and bidirectional axis. Preclinical studies suggest that VNS can alter gut microbial composition, potentially by modulating gastrointestinal motility, secretion, and barrier function. Conversely, as discussed in Section 2.1, microbial metabolites can activate vagal afferents, creating a feedback loop that links peripheral microbial ecology to CNS function. This VNS–microbiota interplay opens new avenues for therapeutic intervention, suggesting that VNS may exert some of its central effects indirectly by reshaping the gut ecosystem, while microbiome-targeted therapies may depend on an intact vagal pathway for their central efficacy (Faraji et al., 2025b).

Therefore, VNS should be viewed not merely as an anti-inflammatory device, but as a multimodal neuromodulator capable of influencing neurotransmitter balance, neuroplasticity, and the MGBA at multiple levels. This broader perspective aligns VNS with the concept of “network pharmacology,” where therapeutic benefit arises from modulating multiple nodes within a complex biological system rather than targeting a single molecule.

### 2.4 Intestinal barrier and blood–brain barrier

These two barriers: the intestinal barrier (intestinal epithelial barrier) and the BBB make up the important defenses against deleterious substances and keep their corresponding microenvironments stable. These two barriers are closely related to one another in the MGBA. Hyperpermeability of intestines, commonly known as leaky gut, is considered to be one of the major triggering conditions that cause systemic inflammation, then breaks the BBB, and finally undermines the neurological well-being (Beltran-Velasco and Clemente-Suárez, 2025).

High intestinal permeability is the start of systemic inflammation. In case of gut dysbiosis or intestinal mucosa damage, tight junction protein (e.g., occludin and claudin) expression or functionalities between intestinal epithelial cells are reduced resulting in impaired barrier performance (Jiang et al., 2024). This breach of intestinal lumen allows the bacteria with their toxic substances (endotoxin LPS) and other pro-inflammatory measures of bacteria to enter the portal vein and then the systemic circulation (Ait Chait et al., 2020). Such molecules as LPS are strong inflammatory agonists (Beltran-Velasco and Clemente-Suárez, 2025; Sittipo et al., 2022), and by binding pattern recognition receptors (e.g., TLR4) on immune cells such as macrophage stimulate downstream signaling pathways. This results in the liberation of pro-inflammatory cytokines in the form of TNF-alpha, interleukin-6 (IL-6) and IL-1 beta, which further promotes a long term low-grade systemic inflammatory condition (Beltran-Velasco and Clemente-Suárez, 2025).

Systemic inflammation directly attacks and disrupts the BBB. The function of the BBB is highly depends on the tightly organized tight junctions between brain endothelial cells (Beltran-Velasco and Clemente-Suárez, 2025). Pro-inflammatory cytokines circulating in the bloodstream (particularly TNF-α and IL-1β) can reach the brain via the vasculature. These cytokines trigger inflammatory signaling pathways in brain endothelial cells, including the nuclear factor κB (NF-κB) pathway, which results in the loss and de-expression of major tight junction proteins, including claudin-5 and occlusin. Such structural perturbation will lead to a rise in BBB permeability (Beltran-Velasco and Clemente-Suárez, 2025). Experimental studies have shown that mice colonized with dysbiotic microbiota from patients with autoimmune encephalitis exhibit compromised BBB integrity, evidenced by increased Evans blue dye leakage and decreased cerebal expression of tight junction proteins ZO-1 and claudin-5 (Gong et al., 2024). After BBB disruption, neurotoxic substances previously excluded in the periphery—including elevated LPS and cytokines—along with peripheral immune cells, infiltrate the brain parenchyma (Liu X. et al., 2025; Stolzer et al., 2023). This invasion activates resident brain immune cells, microglia, initiating and amplifying neuroinflammatory responses. Neuroinflammation is a core pathological hallmark of neurodegenerative diseases such as AD and PD (Beltran-Velasco and Clemente-Suárez, 2025).

Gut microbiota metabolites exert bidirectional regulatory effects in the gut-brain barrier axis (Beltran-Velasco and Clemente-Suárez, 2025). On one hand, dysbiotic microbiota may overproduce harmful metabolites, such as certain secondary BAs, which exacerbate systemic inflammation. On the other hand, SCFAs such as butyrate and propionate—produced by beneficial bacteria fermenting dietary fiber—have been shown to protect both the intestinal barriers and BBB (Beltran-Velasco and Clemente-Suárez, 2025; Liu X. et al., 2025). SCFAs enhance barrier integrity by upregulating tight junction proteins (e.g., occludin, ZO-1) across both barriers through mechanisms including inhibition of HDACs and activation of specific GPCRs (Lee et al., 2024; Shahbazi et al., 2023). Animal experiments have shown that SCFA supplementation minimizes intestinal harm and leakage of the BBB in animals with models of sepsis-linked encephalopathy, and that it goes along with cognitive performance improvement (Cryan et al., 2019). These observations indicate that it is important to keep the gut microbiota in a healthy condition and ensure the sufficient production of SCFA are two essential pharmacological interventions that help to ensure the stability of the gut-brain barrier axis (Liu X. et al., 2025).

## 3 Pharmacological targets along the gut–brain axis

Having established the key physiological pathways through which the gut microbiota communicates with the brain—including microbial metabolites, neuroimmune regulation, vagal signaling, and barrier function—we now turn to the pharmacological strategies designed to modulate these pathways for therapeutic benefit.

### 3.1 Microbiota composition modulators

Therapeutic strategies aimed at modulating gut microbiota composition represent a cornerstone of pharmacological manipulation of the MGBA (Airola et al., 2023). Different microbiota modulators also change microbial structure and function thus affecting intestinal homeostasis and indirectly altering immune, metabolic, and neural signaling pathways (Frias et al., 2025). This kind of modulation has the promise of relieving pathological processes associated with neurological disorders. Another major category of modulators will consist of gut-targeted antibiotics, an example of which is rifaximin (Patel et al., 2022). Since rifaximin is a locally-acting, minimally-absorbable oral antibiotic, the rationalization of the gut microbiome by rifaximin to a desired eubiotic regulatory state is possible; specifically, through selective silencing of potentially harmful bacteria and inhibition of the inflammatory stimuli they generate (Hong et al., 2022). This method achieves microbial balance and does not interfere with diversity significantly. Both clinical and mechanistic research reveal that rifaxib decreases the levels of pro-inflammatory cytokines and systemic inflammation and enhances intestinal barrier integrity (Hong et al., 2022). Rifaximin induces the improvement of cognitive and neurological impairment of MGBA dysfunction by inhibiting the translocation of bacterial components and inflammatory molecules into the circulation (Lin et al., 2025). Cognitive function gets better with rifaximin in experimental models and reduces systemic inflammatory markers and reinforces intestinal barrier functionality, indicating that rifaximin might have neuroprotective effects as a gut microbiota modulator (Li et al., 2021).

One popular category of microbiome-based interventions to restore or maintain a healthy gut microbiota composition is represented by probiotics, prebiotics, and synbiotics (Wadan et al., 2025). Extensive literature reviews have shown that these therapies enhance gut barrier mechanisms, fine-tune host immune activities, and reduce inflammatory mechanisms through immune-neural pathways by fostering the development of beneficial strains, including Lactobacillus and Bifidobacterium, boosting SCFA synthesis, and providing beneficial metabolites (Xu and Lu, 2025). Probiotics also engage in anti-inflammatory signaling, inhibition of pro-inflammatory cytokine secretion, and activation of regulatory T cells, which are activated by probiotics in animal models of neuroinflammation and neurodegenerative disease (Xu and Lu, 2025). Also, synbiotics which are probiotics mixed with prebiotics to synergize the survival and activity of bacteria are being considered as having the capacity to positively regulate the gut microbiota and promote the neurologic health of the host (Connell et al., 2026).

Bacteriophage therapy provides a more specific approach to microbial modulation, unlike antibiotics and probiotics. Bacteriophages are virus specifically infecting and lysing bacterial species, permitting the specific containment of microbial host levels by selective elimination of pathogenic or immunogenic strains and avoids causing an overall disruption of microbial communities (Kuang et al., 2026). Recent review notes that intestinal bacteriophages as the essential element of the gut virome which play a major role in determining the microbial structure in their interactions with the bacteria (Shen et al., 2025; Yahya et al., 2025). Despite all of the above being a nascent area and the inability to clinically apply phage to neurological diseases, it has begun to show promise that phage-mediated elimination of pathogenic bacterial strains can treat inflammatory bowel diseases and indirectly affect immunity. The findings offer a theoretical basis for the future use of phages as a specific microbiome modulator in neurological diseases (Zhou et al., 2025). Nonetheless, issues related to targeting accuracy, consistency in the gastrointestinal tract, and safety in the long term still persist, which makes it necessary to conduct additional mechanistic research and clinical trials (Kuang et al., 2026).

Lastly, FMT has emerged as a popular topic in recent times as an explicit method of rectifying the gut microbiome (Macintosh and Holsinger, 2023). Transplantation of fecal microbiota of healthy hosts into the intestines of patients with FMT quickly rearranges the microbiota composition, increasing microbial diversity, and has a potentially promising impact on neurological performance in some neuropathological disorders (Zhang et al., 2026). As an example, FMT has demonstrated the capacity to better the conduct, dim less inflammatory molecules, and modify gut microbial communities in experimental models of cognitive impairment and neuropsychiatric manifestations related to chronic inflammatory conditions (Baldi et al., 2021; Loh et al., 2024). However, FMT is a topic of concern in pharmacology. Such concerns as the choice of donor microbiota, standardization of treatment, the risks of pathogen spreading, and the ambiguity about the effectiveness in the future require a high level of rigorous clinical and ethical consideration prior to its systematic use (Zhang et al., 2026; Baldi et al., 2021; Loh et al., 2024).

### 3.2 Microbial metabolites as drug targets

While modulating microbial composition represents one approach to GBA-targeted therapy, an alternative and increasingly prominent strategy focuses on the functional output of the microbiota—specifically, the neuroactive metabolites that serve as the direct chemical mediators of gut-brain communication.

The metabolites produced by gut microbiota serve as key chemical mediators in the MGBA, which is attractive and available as a target drug in the treatment of neurological disorders. Recently, their intricate mechanism of action and their role has been clarified, leading to the development of specific intervention plans in relation to these metabolites. In particular, SCFAs (butyrate and propionate) have a significant pharmacological role among them (Lee et al., 2024). As detailed in Section 2.1, SCFAs exert their effects through multiple mechanisms including HDAC inhibition (Li Y. et al., 2026), GPR41/43 receptor activation (Ding et al., 2025) and modulation of microglial function. Building on these mechanistic insights, recent studies have focused on harnessing SCFAs therapeutically. For instance, in AD models, restoring SCFA levels reduces neuroinflammation and improves cognitive function, partially via regulation of the complement C1q pathway (Liu X. et al., 2025). In ASD models, colon-targeted butyrate delivery systems have been shown to modulate serotonin synthesis and gut-brain neural signaling, improving behavioral outcomes (Li W. et al., 2025). These results prove that production and release of local SCFA, which is regulated through prodrug or dietary intervention programs, is a critical and achievable therapy.

Tryptophan-kynurenine (TRP-KYN) nexus is one of the pharmacological nexuses that control neuroexcitatory/inhibitory balance and immunological parameters (Li Y. et al., 2026). The gut microbiota and the host cells decompose tryptophan to neuroactive kynurenine derivatives, and the ratio of kynurenine QUIN (KYNA) to QUIN is an essential factor (Sittipo et al., 2022). KYNA has an antagonistic effect on α7-nicotinic acetylcholine receptors and NMDA receptors, whereas QUIN is an agonist of NMDA receptors, which stimulates excitotoxicity and neuroinflammation (Pocivavsek et al., 2024). The break of this balance in the main depressive disorder and stress-related models specified by lowering KYNA/KYN ratios or raising QUIN levels is directly related to the pathogenesis of the disease (Zhang H. et al., 2025). The pharmacological intervention, therefore, tries to reestablish this balance; these are kynurenine-3-monooxygenase-inhibitors to promote the production of KYNA, and KYNA analog and precursors (Li et al., 2024). Interestingly, a preclinical study has demonstrated that the antidepressant kynurenine antagonist, memantine, selectively increases cortical KYNA levels, leading to its putative therapeutic action being a kynurenine pathway modulator (Grant et al., 2026). Moreover, the direct supplementation of some probiotics (e.g., *Lactobacillus lactis* Lp815) does not only elevate the level of peripheral GABA, but also enhances tryptophan metabolism, suggesting a multi-target heterologous therapeutic potential (Grant et al., 2026).

Metabolites produced by the gut microbiota by the alteration of the primary BAs, known as secondary BAs, have special neuroprotective pharmacological actions (Lee et al., 2024). One of the best-studied representatives of these is the TUDCA. TUDCA penetrates the BBB and has anti-apoptotic, antioxidant, and anti-inflammatory effects in the mitochondria via the activation of farnesoid X receptors (FXR) and TGR5 receptors (Hurley et al., 2022). These properties have also illustrated obvious neuroprotective effects in preclinical models of various neurodegenerative diseases, including PD, Huntington’s disease and amyotrophic lateral sclerosis (ALS) and have progressed to several clinical trials (Hurley et al., 2022; Ji et al., 2025). It has even been extended by clinical observational studies to be therapeutic. As an example, high serum levels of total bile acid are positively correlated with better outcomes of ischemic stroke patients who do not receive anticoagulant treatment (Wang et al., 2024). These results imply that bile acid pool composition is a targetable neuroprotective therapeutic approach where bile acid analogs or microbiota transplantation or specific probiotic interventions can be used to modulate the composition of the bile acid pool.

Lastly, GABA and serotonic 5-HT precursors, as well as precursors of their microbial synthesizers, present direct opportunities as a new therapeutic agent in neuropsychiatric disorders. The major neurotransmitter is GABA, which is the main inhibitory neurotransmitter (Sittipo et al., 2022). Certain species of the genus Bacteroides and *Lactobacillus plantarum* Lp815 have been shown to be capable of synthesizing GABA *in vivo* in the gut (Yousuf et al., 2025). A randomized controlled trial was conducted in 2026 proving that continuous insomnia and anxiety symptom reductions under *Lactobacillus plantarum* Lp815 at 6 weeks were also accompanied by urinary GABA level increase (Grant et al., 2026). Moreover, increases in GABA levels negatively correlated with symptom severity, providing direct human evidence for the “strain-metabolite-therapeutic effect” chain. This breakthrough marks the transition of “psychoactive probiotics” from concept to clinical application—systemically regulating host physiology by orally supplementing of strains producing specific neuroactive metabolites. Similarly, enhancing tryptophan bioavailability of tryptophan (a precursor to 5-HT) or supplementing with strains that produce serotonin precursors such as 5-hydroxytryptophan offers MGBA-targeted alternative or adjunctive therapies for emotional disorders, including depression and anxiety (Yousuf et al., 2025; Dziedziak et al., 2025).

Despite their promising effects, a key pharmacological challenge for SCFAs, particularly butyrate, is their rapid metabolism and poor pharmacokinetic profile. To achieve therapeutic concentrations in the CNS, novel delivery strategies are being explored (Dalile et al., 2019; Silva et al., 2020). To overcome these limitations and achieve therapeutically relevant CNS exposure, recent research has focused on novel delivery strategies. One approach involves the development of prodrug formulations that release butyrate in the distal gut, such as butyrylated starches, thereby leveraging endogenous gut–brain signaling pathways rather than relying on direct brain penetration (Koh et al., 2016). Another promising avenue is the design of nanocarriers and lipophilic analogs to enhance stability, systemic half-life, and BBB permeability. Nanotechnology-based vehicles, including polymeric nanoparticles and lipid-based systems, have been shown to improve transport across the BBB and increase brain bioavailability of small molecules (Saraiva et al., 2016). The field is moving from simply observing SCFA effects to engineering their delivery for CNS indications.

### 3.3 Immune-inflammatory signaling modulators

Beyond targeting microbial metabolites, another major arm of GBA-directed pharmacotherapy aims to directly modulate the host immune-inflammatory response, which serves as a critical downstream effector of microbial signals and a key driver of neuropathology.

Targeting neuroinflammatory signaling pathways represents a core therapeutic strategy for neurodegenerative diseases such as Alzheimer’s and PD. Among the key immunomodulatory agents, TNF-α inhibitors, IL-6 blockers, and NLRP3 inflammasome inhibitors have shown significant therapeutic potential (Alemán-Villa et al., 2025).

TNF-α is a key cytokine driving neuroinflammation and neuronal injury (Xie et al., 2024). Traditional large-molecule monoclonal antibodies struggle to effectively cross the BBB, restricting their therapeutic application in CNS disorders. Recently, novel inhibitors like nanobodies have emerged as a research hotspot. Single-domain nanobodies derived from camelids (approximately 12 kDa) have approximately one-tenth the molecular weight of conventional antibodies, which reduces immunogenicity and significantly enhances tissue penetration (Yin et al., 2025). A 2025 study reported humanized TNF-α inhibitory nanobodies (TNFI-Nbs) capable of efficiently neutralizing TNF-α at picomolar concentrations, demonstrating activity surpassing traditional antibodies (Yin et al., 2025). Crucially, engineered strategies have further enhanced nanobody functions. For instance, fusing the extracellular domain of the TNF receptor with an antibody fragment targeting the transferrin receptor created a dual-functional molecule capable of actively crossing the BBB via receptor-mediated endocytosis (Jagadeesan et al., 2024). A 2024 animal study confirmed that this brain-penetrant TNF-α inhibitor successfully entered the brains of AD model mice, significantly modulating the hippocampal proteome associated with microglial function and reducing β-amyloid plaques (Jagadeesan et al., 2024). These advances open new pathways for precisely targeting central TNF-α (Goshi et al., 2025).

IL-6 is another crucial pro-inflammatory factor, signaling primarily occurs via membrane-bound receptors (classical signaling) and soluble receptors (trans signaling) (Alemán-Villa et al., 2025; Siuchnińska et al., 2025). Clinically-used IL-6 blockers are primarily monoclonal antibodies, such as siltuximab targeting IL-6 itself and tocilizumab targeting its receptor. Additionally, small-molecule JAK inhibitors (e.g., tofacitinib, baricitinib) block the JAK–STAT pathway, simultaneously inhibiting downstream signaling of multiple cytokines, including IL-6 (McElvaney et al., 2021). However, compared to their widespread use in peripheral immune diseases like rheumatoid arthritis, clinical exploration of IL-6/JAK–STAT pathway inhibitors in neurological disorders remains in its early stages (McElvaney et al., 2021). The most difficult part is the task of determining the balance between systemic immunosuppression on the one hand and specific anti-inflammatory action on the other in the CNS (Shan et al., 2024).

The NLRP3 inflammasome is a central node of sensitizing a variety of danger signals, promoting the maturation and release of IL-18 and IL-12 to become an important initiator of chronic neuroinflammation in neurodegenerative diseases (Xu et al., 2025). As a result, NLRP3 direct inhibition can be regarded as a promising approach to prevent the inflammatory cascade on a very high level (Mustafa et al., 2025). Recent research has focused on developing small-molecule inhibitors with enhanced BBB penetration. For instance, NT-0796 is a prodrug with high lipophilicity and brain permeability (Digby et al., 2026). Upon entering the brain parenchyma, it is primarily activated by glia-derived carboxylesterase-1 to produce potent active metabolite NDT-19795, which exerts localized inhibitory effects at the lesion site (Digby et al., 2026). This ingenious “brain-activated” prodrug design enhances both targeting accuracy and safety (Digby et al., 2026). Additionally, multiple novel candidate drugs are under development. In 2026, ISM8969—an orally administered, BBB-penetrating candidate, received authorization for CNS disease development, with preclinical studies demonstrating significant anti-inflammatory activity. Concurrently, derivatives of the natural product donglingcao A have also been shown to effectively inhibit NLRP3 pathway activation (Ou et al., 2025).

The development of brain-penetrant TNF-α inhibitors, including engineered nanobodies fused to transferrin receptor (TfR)-targeting antibodies, exemplifies a fundamental pharmacological principle: the exploitation of endogenous transport systems to overcome the BBB. Rather than relying solely on molecular potency, the therapeutic success of TNF-α inhibition in the CNS critically depends on efficient brain delivery (Pardridge, 2012; Pardridge, 2019). Receptor-mediated transcytosis (RMT), particularly via the TfR pathway, has emerged as a leading strategy for enhancing CNS exposure of biologics. By coupling therapeutic agents to ligands or antibodies targeting BBB transport receptors, macromolecules can be actively shuttled across endothelial cells into brain parenchyma (Niewoehner et al., 2014; Yu et al., 2011). This approach represents a significant advance for biologic therapies aimed at modulating neuroinflammation. Importantly, RMT-based delivery systems are not limited to TNF-α inhibitors. Similar engineering strategies may be applied to other macromolecular gut–brain axis-targeted agents, including peptides, cytokine modulators, and nanobody-based therapeutics (Johnsen et al., 2019).

### 3.4 Neural pathway modulators

In parallel with pharmacological modulation of immune signaling, direct targeting of neural pathways—particularly the VN—offers a distinct and complementary approach to harnessing the MGBA for therapeutic benefit.

VNS activates the VN through physical or electrical stimulation, representing the most direct method to regulate the central autonomic nervous system (CAP) (Liu J. et al., 2025). Initially used to treat epilepsy, recent studies have confirmed its significant anti-inflammatory, analgesic, and mood-regulating effects across various immune-mediated diseases such as rheumatoid arthritis and Crohn’s disease (Liu J. et al., 2025; Shen et al., 2024). The core anti-inflammatory mechanism of VNS heavily relies on CAP activation: stimulation signals travel via vagal afferent fibers to the brainstem, where they are integrated before influencing peripheral immune organs like the spleen through efferent fibers (Chen et al., 2022; Leem et al., 2025). In the spleen, norepinephrine release induces specific T cells to synthesize and release acetylcholine. This acetylcholine then binds to α7 nicotinic acetylcholine receptors (α7nAChR) on immune cells like macrophages (Liu Y. et al., 2025). This connection enables the intracellular route of JAK2/STAT3 signaling and, at the same time, arrests the major pro-inflammatory transcription factors such as NF-κB (Chen et al., 2022; Li Y. et al., 2025). Finally, VNS causes a considerable reduction in the release of pro-inflammatory cytokines such as tumor necrosis factor-alpha (TNF-α) and interleukin-6 (IL-6) (Liu J. et al., 2025; Li Y. et al., 2025). A 2024 systematic review and meta-analysis further revealed that various VNS modalities [e.g., transcutaneous auricular vagal nerve stimulation (taVNS), implantable VNS (iVNS)] have varying effects on particular inflammatory mediators, including C-reactive protein, IL-10, IL-1β, which implies that modalities of stimulation and the choice of parameter factors are important in therapeutic effectiveness (de Melo et al., 2025). One clinical study also demonstrates that VNS is very effective in improving disease activity indices and lowering inflammatory markers in Crohn’s disease, 12 months of effect of which has been proven (Chen et al., 2022).

In addition to physical neural stimulation, the pharmacological activity of the main nodes of the CAP pathway, in particular α7nAChR, is another important intervention strategy (Sethna et al., 2022; Parente et al., 2024). The agents have a goal of imitating or intensifying the endogenous acetylcholine anti-inflammatory signaling. Studies also reveal that the engagement of α7nAChR not only suppresses the activation of NF-κB and NLRP3 inflammasome but also increases the levels of intestinal tight junction proteins (e.g., occludin, claudin-1) via the AMPK/mTOR autophagy axis (Chen et al., 2022; Leem et al., 2025; Sethna et al., 2022; Shao et al., 2019). This dual effect suppresses inflammation and enhances intestinal epithelial barrier function. Consequently, α7nAChR agonists (e.g., GTS-21, PNU-282987) have demonstrated therapeutic potential in multiple models, including inflammatory bowel disease (Chen et al., 2022; Sethna et al., 2022; Pu et al., 2022). Interestingly, certain drugs already used clinically in other fields exhibit partial efficacy linked to indirect activation of CAP (Chen et al., 2022). For instance, dexmedetomidine, a highly selective α2-adrenergic receptor agonist, improved cognitive function, inhibited hippocampal microglial activation, and reduced systemic inflammatory mediators (TNF-α, IL-6, IL-1β) in an aged mouse model of postoperative cognitive impairment. Notably, these effects were blocked by an α7nAChR-specific antagonist (Chen et al., 2022; Zhang Q. et al., 2025). This suggests that dexmedetomidine may exert neuroprotective effects by indirectly activating the CAP centered on α7nAChRs through a “parasympathetic-like” effect (Zhang Q. et al., 2025).

### 3.5 Barrier-targeting agents

A final, yet critically important, category of GBA-targeted interventions focuses on the structural barriers that separate the gut lumen from the circulation and the circulation from the brain—namely, the intestinal barrier and the BBB.

Tight junction modulators are designed to precisely regulate the tight junction protein complexes between barrier epithelial and endothelial cells. Claudin-5 (CLDN5), the core tight junction protein of the BBB, has emerged as a key therapeutic target (Hashimoto and Campbell, 2020). Current research focus is shifting from traditional non-specific permeability enhancers toward mechanism-specific, targeted modulators (Brunner et al., 2021). For instance, structural modeling facilitated the design of a novel short peptide, f1-C5C2, derived from the extracellular domain of CLDN5. This peptide transiently and reversibly increases barrier permeability in *in vitro* BBB models, creating a potential “time window” for administering therapeutic drugs such as glucose for treating glucose transporter 1 deficiency (Hashimoto and Campbell, 2020). The use of small-molecule inhibitors, such as compound M01, that bind specifically to the extracellular domain of CLDN5 and impairs the cell–cell connection and trigger the process of CLDN5 endocytosis is another approach. This causes a temporary BBB in animal models to increase the delivery of chemotherapy drugs to brain tumors (Breitkreuz-Korff et al., 2021). These illustrations show that specific molecular vectors acquired by engineering or selection can not only act uniquely to open the BBB to allow a therapeutic benefit, but that their converse application, tightening up junctions, can recover theoretically be employed to break and restore the barrier integrity.

Zonulin inhibitors are directed to the central pathway of the regulation of abnormal intestinal barrier permeability (Veres-Székely et al., 2023a). Zonulin is one of the most important physiological proteins that control the opening of the tight junctions of the intestines, and its hyper-expression correlates with leaky gut in numerous diseases (Veres-Székely et al., 2023a; Slifer et al., 2021). One of its inhibitors lactazepam, has become a prototype drug in the area (Troisi et al., 2021). Myosin light chain kinase is a downstream signaling pathway antagonized by lactazepam, which stabilizes tight junctions and lowers intestinal permeability in tension of the zonulin receptor (Troisi et al., 2021; Veres-Székely et al., 2023b). Preclinical research supports the conclusion that lactazepam enhances intestinal and systemic inflammatory diseases barrier functions and pathological alleviation in multiple models of intestinal and systemic inflammatory diseases (Slifer et al., 2021; Troisi et al., 2021). Whereas these therapies are originally intended to act in the intestine, with little absorption of a drug into the systemic circulation upon oral administration, it is suggested by some scientists that, due to the role of the zonulin pathway in breaking down bloodbrain barriers, such agonists may have some uses in CNS disease in situations where they are administered by alternative delivery routes or preparations (Veres-Székely et al., 2023b).

**Figure 2:**
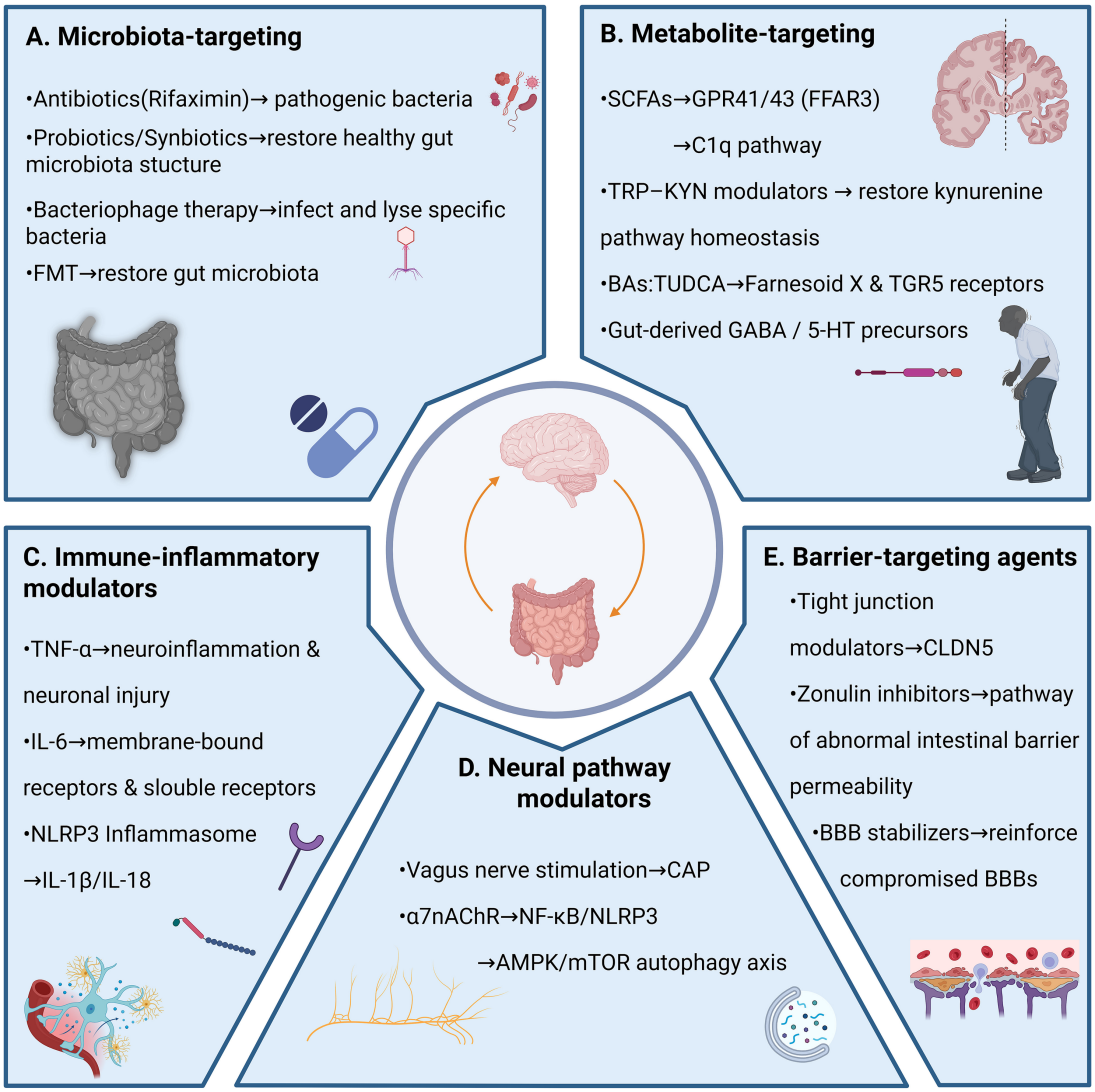
Pharmacological targeting strategies along the GBA. Overview of major therapeutic approaches categorized by their primary site of action within the GBA. Panel **A** (microbiota-directed therapies): strategies aimed at reshaping microbial composition and function, including: (i) antibiotics (e.g., rifaximin) for selective elimination of pathobionts; (ii) probiotics and prebiotics to enhance beneficial taxa and metabolic output; (iii) FMT for comprehensive microbial restoration; and (iv) bacteriophage therapy for targeted depletion of specific bacterial strains. Panel **B** (metabolite-targeted strategies): interventions focused on microbial metabolites and their host receptors, including SCFAs (butyrate, propionate, acetate) acting on GPR41/43, BAs (TUDCA, CDCA) engaging TGR5 and FXR, and tryptophan-kynurenine pathway modulators (IDO1 inhibitors, KYNA analogs). Panel **C** (immune-inflammatory modulators): agents targeting neuroinflammation, including TNF-α inhibitors (nanobodies), IL-6/JAK–STAT pathway blockers (tocilizumab, baricitinib), and NLRP3 inflammasome inhibitors (MCC950, NT-0796). Panel **D** (neural pathway modulators): interventions modulating vagal signaling, including electrical VNS and pharmacological agents targeting the cholinergic anti-inflammatory pathway (α7nAChR agonists such as GTS-21 and PNU-282987). Panel **E** (barrier-targeting agents): strategies to restore intestinal and BBB integrity, including tight junction modulators (claudin-5 regulators), zonulin inhibitors (larazotide acetate), and BBB stabilizers (WNT/β-catenin pathway agonists). Each category offers distinct entry points for pharmacological intervention, with the potential for combination strategies targeting multiple nodes simultaneously. BBB.

BBB stabilizers target having a direct reinforcing effect on damaged BBBs against neuroinflammatory damage. New approaches are better than traditional anti-inflammatories in that they directly regulate homeostatic pathways of BBB endothelial cells (Ding et al., 2023; Binder et al., 2025). Another major advance is to target the Frizzled-4 (FZD4) receptor to reactivate WNT/-catenin pathway that has been shown to play a central role in BBB formation and maintenance (Ding et al., 2023). The group of researchers like L6-F4-2 is a bioengineered FZD4-specific WNT agonist with sub-picomolar affinity that activates protective signaling in BBB endothelial cells powerfully (Ding et al., 2023). In an adult mouse ischemic stroke model, post-stroke administration of L6-F4-2 reversed BBB leakage, reduced cerebral edema and infarct size, and improved neurological function scores (Ding et al., 2023). Furthermore, novel endogenous BBB regulatory targets have been identified, such as the metalloproteinase Meprin β, whose knockout increases tight junction protein expression in cerebral microvessels and reduces BBB permeability (Gindorf et al., 2020; Sugiyama et al., 2023). These discoveries provide novel molecular targets for the development of drugs designed to directly “fortify” and restore BBB integrity (Table 1 and Figure 2).

**Table 1:** Pharmacological agents targeting the gut–brain axis: molecular targets, mechanisms, and translational evidence.

Drug/Intervention	Primary pharmacological target	Key mechanism of action	Disease relevance	Experimental model	Translational status	References
Rifaximin	Gut microbiota composition	Reduces pro-inflammatory taxa; increases SCFA-producing bacteria; lowers endotoxemia	PD, HE, MDD	Animal models; small clinical studies	Approved (non-CNS); repurposing	Sittipo et al. (2022), Gong et al. (2024), and Stolzer et al. (2023)
Probiotics (Lactobacillus, Bifidobacterium)	Microbiota–metabolite axis	↑ SCFAs; ↓ systemic inflammation; modulates HPA axis	AD, MDD, ASD, MS	Animal models; small RCTs	Exploratory clinical	Shahbazi et al. (2023), Airola et al. (2023), Ding et al. (2023), and Binder et al. (2025)
Synbiotics	Microbiota + substrate availability	Enhances microbial metabolic output (SCFAs, tryptophan metabolites)	MDD, ASD	Animal; pilot trials	Early clinical	Shahbazi et al. (2023), Airola et al. (2023), and Frias et al. (2025)
GTS-21	α7nAChR	Activates cholinergic anti-inflammatory pathway (CAP); inhibits NF-κB and NLRP3	IBD, cognitive disorders	Animal models	Preclinical	Pardridge (2012), Johnsen et al. (2019), and Chen et al. (2022)
PNU-282987	α7nAChR	Suppresses microglial activation; enhances barrier integrity	Neuroinflammation	Animal models	Preclinical	Pardridge (2012), Johnsen et al. (2019), and Chen et al. (2022)
Dexmedetomidine	Indirect CAP activation	α2-AR-mediated vagal tone ↑; α7nAChR-dependent anti-inflammatory effects	POCD, stroke	Animal + clinical	Approved (anesthetic)	Pardridge (2012) and Leem et al. (2025)
Tocilizumab	IL-6 receptor	Blocks IL-6-JAK-STAT signaling; reduces neuroinflammation	NMOSD, RA	Clinical trials	Approved	Wang et al. (2024), Koh et al. (2016), and Saraiva et al. (2016)
Sarilumab	IL-6 receptor	Similar IL-6 pathway inhibition	RA, COVID-19	Clinical trials	Approved	Wang et al. (2024), Koh et al. (2016), and Saraiva et al. (2016)
MCC950	NLRP3 inflammasome	Selective inhibition of NLRP3 activation and IL-1β release	AD, PD, MS	Animal models	Preclinical	Xie et al. (2024), Yin et al. (2025), and Jagadeesan et al. (2024)
Larazotide acetate	Zonulin pathway	Restores tight junction integrity; reduces gut permeability	Celiac, neuroinflammation	Animal; phase 2	Phase 2	Parente et al. (2024), Shao et al. (2019), and Pu et al. (2022)

This table summarizes representative pharmacological interventions targeting the gut–brain axis, highlighting their primary molecular targets, core mechanisms of action, disease relevance, and current translational status. Emphasis is placed on immune-inflammatory signaling, microbial metabolite pathways, neural modulation, and barrier integrity.

## 4 Disease-specific pharmacological evidence

The pharmacological strategies outlined above—targeting microbial composition, metabolites, immune signaling, neural pathways, and barrier function—are not disease-specific in principle. Rather, they target shared pathophysiological mechanisms that operate across multiple neurological and psychiatric conditions. In the following section, we examine how these mechanisms manifest in specific disorders and review the clinical evidence for GBA-targeted interventions in each disease context.

### 4.1 Parkinson’s disease

In Parkinson’s disease (PD), MGBA dysregulation, gut microbiota imbalance, and inflammatory pathways have been extensively studied and are increasingly recognized as being associated with α-synuclein pathology, glial activation, and neurodegenerative processes (Korszun-Karbowniczak et al., 2024). PD patients often present with intestinal dysfunction, a reduced abundance of short-chain fatty acid (SCFA)-producing bacteria, and an increased presence of harmful bacteria species (Korszun-Karbowniczak et al., 2024). These alterations are hypothesized to contribute to disease progression through immune, metabolic, and inflammatory pathways. Preclinical evidence indicates that gut dysbiosis exacerbates α-synuclein aggregation and neuroinflammation, while interventions such as probiotics, antibiotics (e.g., rifaximin), or FMT have been reported to partially improve motor and non-motor symptoms and reduce inflammatory markers (Bonnechère et al., 2022; Wadan et al., 2025). Clinical translation, however, remains challenging. For instance, while small open-label trials of probiotics (Lactobacillus and Bifidobacterium strains) have consistently reported improved constipation (a non-motor symptom) in PD patients, their effects on core motor symptoms (UPDRS Part III) remain inconsistent, with some studies showing modest benefit and others showing none (Cryan et al., 2019; Unger et al., 2016).

Pharmacological interventions for PD currently primarily focus on indirectly influencing CNS pathology by modulating the microbiome and metabolic signaling (Pang et al., 2025). Limited clinical evidence, small-scale clinical or open-label studies have investigated the effects of rifaximin on gut inflammation and motor symptoms in PD patients, but no large randomized controlled trials have yet confirmed its efficacy. Preclinical studies demonstrating improvements in gut barrier function, reductions in central inflammation, and attenuation of certain behavioral symptoms (Jeong et al., 2025). Consistent with these animal studies, clinical outcomes of gut-targeted interventions exhibit significant heterogeneity, often requiring individualized treatment strategies based on individual microbial profiles and long-term monitoring (Zhou and Dettmer, 2026). This heterogeneity likely stems from several pharmacological factors: (1) strain specificity: not all probiotics produce equal levels of neuroactive metabolites (e.g., GABA, SCFAs) (Cryan et al., 2019; Dalile et al., 2019); (2) disease stage: gut-derived pathology may be more relevant in early, prodromal PD, whereas advanced disease may be driven by CNS-autonomous processes (Houser and Tansey, 2017); (3) outcome measures: constipation improvement may reflect local gut effects (motility), whereas motor improvement would require systemic immune modulation or vagal signaling, which may require higher or more sustained dosing. The antibiotic rifaximin has shown promise in reducing peripheral inflammatory markers in PD, but its inability to cross the BBB limits its effects to peripheral detoxification, underscoring that GBA-targeted therapies primarily act by “cleaning the periphery” rather than directly targeting the CNS (Pardridge, 2012; Sampson et al., 2016).

A major translational barrier is the lack of biomarkers to identify patients who are “gut-driven.” Future pharmacological studies should stratify PD patients based on baseline gut permeability (e.g., serum zonulin levels) or microbial signatures, and employ postbiotic formulations (e.g., encapsulated butyrate) rather than live organisms to ensure consistent pharmacokinetics and bypass viability concerns (Cryan et al., 2019; Fasano, 2012; Scheffler et al., 2018).

It is important to note that while rifaximin represents a useful example of a gut-targeted antibiotic strategy, the therapeutic landscape for PD is considerably broader. Clinical studies have demonstrated that supplementation with specific probiotic formulations, particularly those containing Lactobacillus and Bifidobacterium strains, can significantly improve gastrointestinal dysfunction, including constipation, in PD patients (Barichella et al., 2016). In some cohorts, probiotics have also been associated with modest improvements in motor symptom scores. Mechanistically, these effects are thought to be linked to restoration of SCFA production, improvement of gut barrier integrity, and modulation of systemic inflammatory mediators (Unger et al., 2016). Beyond probiotics, FMT—discussed in Section 3.1 as a strategy for comprehensive microbial restoration—has recently been explored in PD. Preliminary studies suggest that FMT may ameliorate both gastrointestinal and motor symptoms in PD patients, although robust randomized controlled trials remain limited and patient responses are highly variable (Xue et al., 2020). Notably, substantial heterogeneity in patient responses to microbiome-modulating interventions has been observed. This variability underscores the need for precision medicine approaches, potentially guided by baseline microbial profiling and metabolomic signatures (Cryan et al., 2019).

### 4.2 Alzheimer’s disease

While PD exemplifies the role of gut-derived signals in α-synuclein pathology and motor dysfunction, Alzheimer’s disease (AD) presents a distinct but equally compelling case for GBA involvement, centered on systemic inflammation, amyloid pathology, and BBB integrity.

1. The MGBA pathology in AD is closely associated with systemic inflammation and disruption of the BBB. Epidemiological and clinical studies suggest that *Helicobacter pylori* infection is an extrinsic risk factor, which increases the rate of progression in tau pathology and aggravates cognitive decline via more gut microbiota dysbiosis, facilitating the occurrence of BBB disruption (as shown by the increasing levels of MMP-9 in cerebrospinal fluid), and neuroinflammation (Li J. et al., 2026). However, causality remains debated. Further data on animal models indicate that some individual dietary risks factors such as carbon polymers formed during food processing, undermine the intestinal wall, as a result of which LPS is transported into the bloodstream (Aijaz et al., 2025). Subsequently, neuroinflammation and synaptic dysfunction in the brain are induced in the LPS-TLR4-NF-XB-pathway through the circulation of LPS (Qi et al., 2026). As per therapeutic interventions, there are no currently approved AD drugs whose mechanism of action is a direct MGBA. Nevertheless, preclinical evidence suggests a significant amount of evidence that supports microbiota modulation with the use of probiotics, FMT, or a particular antibiotic (e.g., rifaximin Ismeurt-Walmsley et al., 2025). Regardless, human clinical studies have largely found small effects on inflammatory but inconsistent and weak effects on core cognitive outcomes, regardless of animal model results, suggesting that it is difficult to extrapolate results of animal models to the multifaceted pathology of human AD (Loh et al., 2024).

Chronic peripheral infections and systemic inflammation are increasingly implicated in AD pathophysiology. Epidemiological studies have linked *Helicobacter pylori* infection and periodontal pathogens with accelerated cognitive decline and increased dementia risk (Kamer et al., 2012; Beydoun et al., 2013). In particular, *Porphyromonas gingivalis* and its toxic proteases (gingipains) have been detected in AD brains, suggesting a mechanistic connection between peripheral infection and neurodegeneration (Dominy et al., 2019). Experimental evidence provides pharmacological plausibility for this association. In animal models, systemic LPS administration induces sustained neuroinflammation, synaptic dysfunction, and Aβ accumulation, largely mediated through TLR4–NF-κB signaling pathways (Qin et al., 2007). These findings support the hypothesis that targeting peripheral immune activation may attenuate CNS pathology. Despite this rationale, clinical translation has proven challenging. Large randomized controlled trials evaluating anti-inflammatory agents, including NSAIDs, have largely failed to demonstrate efficacy in patients with established AD (Aisen et al., 2003). This discrepancy underscores a fundamental pharmacological principle: therapeutic timing. Preclinical studies typically intervene prior to or during early pathological stages, whereas clinical trials enroll patients with advanced neurodegeneration, where downstream CNS cascades may be less reversible (Perry and Holmes, 2014; Cummings et al., 2022). Microbiome-targeted strategies such as probiotics have demonstrated modest but statistically significant cognitive benefits in some studies and meta-analyses (Zhu et al., 2021). Proposed mechanisms include enhanced production of SCFAs, attenuation of systemic inflammation, and reduction of circulating LPS levels. SCFAs, particularly butyrate, are known to modulate microglial activation and neuroinflammatory responses, providing a mechanistic bridge between gut microbiota and CNS immune homeostasis (Dalile et al., 2019; Erny et al., 2015). However, critical pharmacological uncertainties remain unresolved. Dose–response relationships are poorly defined, and it is unclear whether conventional oral probiotic formulations can achieve CNS-relevant metabolic or immunomodulatory effects. The absence of robust pharmacokinetic–pharmacodynamic (PK–PD) data continues to limit the clinical translation of microbiome-based interventions in AD.

### 4.3 Multiple sclerosis

Having examined neurodegenerative disorders characterized by protein aggregation (PD and AD), we now turn to multiple sclerosis (MS), where the GBA contributes primarily through modulation of autoimmune responses and peripheral immune cell trafficking.

The major signs and symptoms of MS are the autoimmune-mediated CNS inflammation and demyelination. Research on the MGBA shows that MS patients often have altered gut microbiota, characterized by increased pro-inflammatory bacteria and decreased anti-inflammatory bacteria. These changes correlate with peripheral immune activation and an enhanced pro-inflammatory T-cell phenotype. Preclinical models demonstrate that gut microbiota significantly alters the Th17/Treg balance and alleviates central inflammation, suggesting gut microbiota regulation may serve as a promising pharmacological intervention pathway for MS (Omarova and Boyko, 2023; Zang et al., 2023).

Current clinical MS drugs (e.g., interferons, anti-CD20 antibodies) do not directly target the MGBA. However, some studies have investigated probiotics and microbiome modulation as adjunct strategies to decrease inflammatory relapse rates. Animal studies consistently show that microbiota intervention can reduce CNS inflammation and demyelination. Clinical evidence remains in its early stages, primarily consisting of small-scale, short-term trials, and requires more rigorously designed studies for validation (Zang et al., 2023).

### 4.4 Autism spectrum disorder

Beyond autoimmune demyelination, the MGBA also plays a critical role in neurodevelopmental conditions such as autism spectrum disorder (ASD), where microbial metabolites influence neurotransmitter balance and synaptic function during critical developmental windows.

The MGBA mechanisms underlying ASD involve dysbiosis of the gut microbiota and its metabolites (such as SCFAs and phenolic compounds) which disrupt BBB permeability, trigger neuroinflammation, and disturb key neurotransmitter systems, particularly the glutamate/GABA excitatory-inhibitory balance (Bu et al., 2025; Zhang Z. et al., 2025). Preclinical models provide compelling causal evidence: in propionic acid-induced ASD-like mouse models, targeted FMT using Lactobacillus-rich donors not only restored gut microbiota but also directly normalized Glu/GABA ratios and neuronal electrophysiology in the prefrontal cortex, thereby improving social behavior. However, clinical evidence mainly comes from small-scale open-label trials or case series (He et al., 2026). While FMT and specific probiotics (e.g., *Lactobacillus rhamnosus*) show potential in improving gastrointestinal symptoms and behavioral issues in some pediatric patients, their efficacy varies widely among individuals (Bu et al., 2025). This variability partly reflects the high personalization of the human microbiome and the inherent heterogeneity of ASD as a disorder. Consequently, conclusions drawn from relatively homogeneous animal models are difficult to directly translate into universally effective clinical protocols.

The propionic acid (PPA)-induced rodent model has yielded important mechanistic insights into gut–brain interactions relevant to autism spectrum disorder (ASD). Acute intraventricular administration of PPA reproduces ASD-like behaviors, mitochondrial dysfunction, and neuroinflammatory responses, supporting the concept that microbial-derived metabolites can directly influence CNS function (MacFabe et al., 2007). Furthermore, preclinical microbiome manipulation studies demonstrate that FMT or colonization with specific bacterial taxa can modulate social behavior and normalize neurotransmitter balance, including restoration of the prefrontal cortex Glu/GABA ratio (Hsiao et al., 2013). These findings suggest that microbial metabolites may regulate CNS excitatory–inhibitory homeostasis. However, a critical translational limitation must be acknowledged. The PPA model represents an acute, high-dose metabolite challenge, which may inadequately reflect the chronic, low-grade metabolic dysregulation hypothesized in human ASD. Consequently, extrapolation of pharmacological relevance requires caution. Clinical investigations of FMT in children with ASD have reported improvements in both gastrointestinal (GI) and behavioral symptoms, particularly in open-label and non-blinded trials (Kang et al., 2017; Kang et al., 2019). While encouraging, these studies lack rigorous placebo control, and behavioral outcomes are inherently susceptible to expectancy and placebo effects. In addition, the marked heterogeneity of ASD—including idiopathic and syndromic forms—further complicates interpretation of therapeutic responses.

### 4.5 Depression/Anxiety

The influence of gut microbiota on neurotransmitter systems extends beyond neurodevelopment to mood regulation, as evidenced by the growing body of research linking the MGBA to depression and anxiety disorders.

Depression and anxiety, as neuropsychiatric disorders, are closely associated with chronic low-grade inflammation, neurotransmitter imbalances, and abnormal stress responses (Zang et al., 2023). Clinical studies on the MGBA indicates that individuals with depression and anxiety often have reduced gut microbiota diversity and an increased abundance of pro-inflammatory bacteria. Additionally, a deficiency in SCFAs may impair neurotransmitter synthesis and inflammatory regulation. Gut-brain signaling influences emotional states through immune, neural, and endocrine pathways (Zhang et al., 2026).

At the pharmacological level, preclinical evidence demonstrates that certain probiotics (termed psychobiotics) have demonstrated potential to alleviate depressive and anxious behaviors and reduce inflammatory markers in animal studies and small-scale clinical trials. However, robust evidence from large-scale randomized controlled trials (RCTs) remains limited (Kakinaka and Umeno, 2020). Some probiotic and prebiotic interventions have shown improvements in depression rating scales like the HAM-D, though the magnitude of effect and long-term efficacy remain controversial (Kakinaka and Umeno, 2020). Furthermore, existing antidepressants such as SSRIs/SNRIs may exert partial effects by indirectly influencing gut signaling (e.g., 5-HT precursors), but these do not constitute drugs targeting the MGBA specifically (Tan et al., 2021).

### 4.6 Stroke

Finally, the MGBA has emerged as a modulator of acute neurological injury, with post-stroke dysbiosis influencing BBB integrity and neuroinflammatory outcomes—a rapidly evolving area with distinct therapeutic implications.

**Figure 3:**
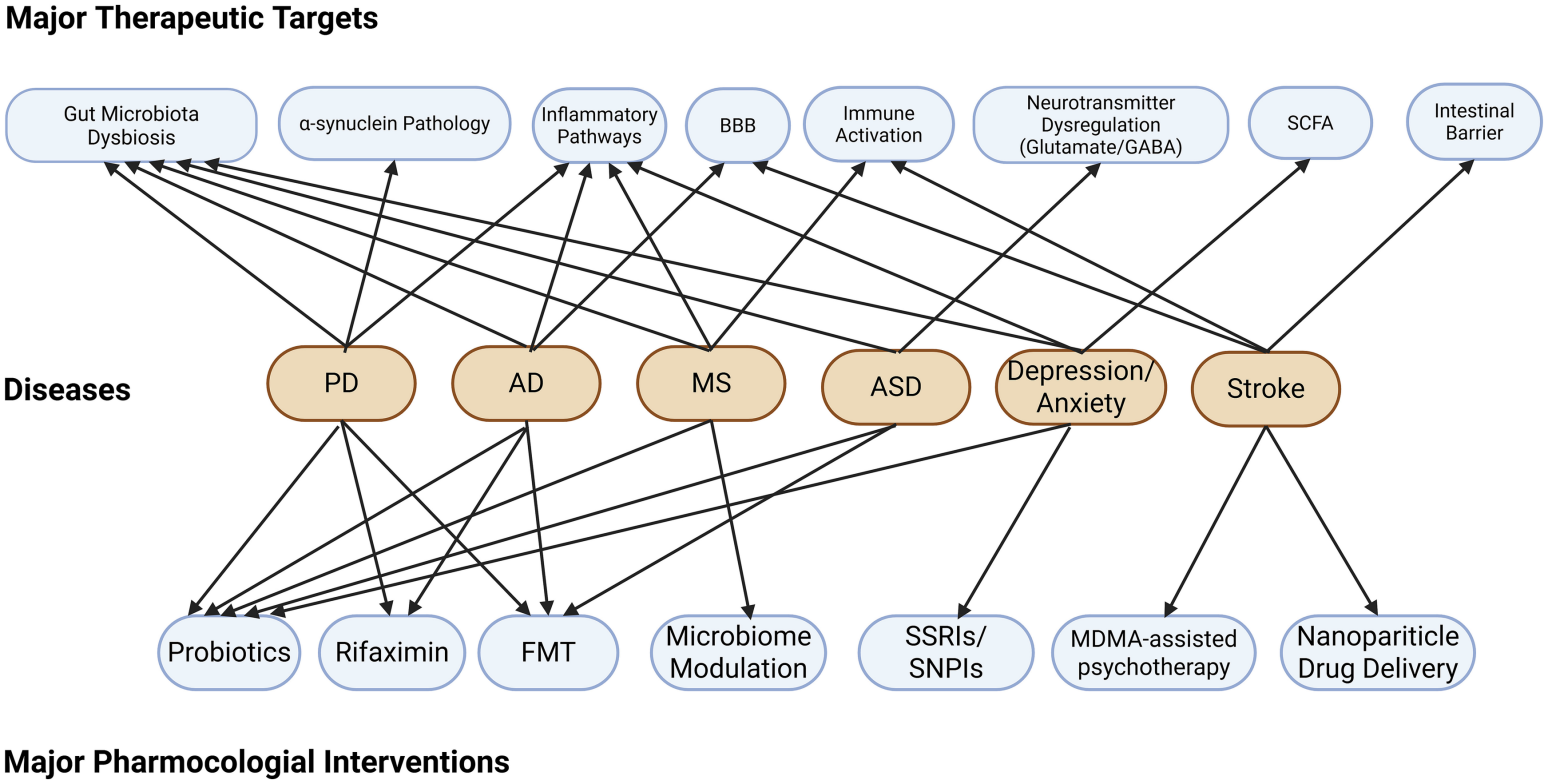
Disease-target-drug network diagram: shared mechanisms and therapeutic opportunities. This schematic illustrates representative and dominant pathophysiological targets linking gut-brain axis (GBA) dysfunction to neurological and psychiatric disorders, rather than providing an exhaustive depiction of all possible interactions. The diagram is organized to highlight the concept of shared mechanisms across diseases and the potential for target-oriented rather than disease-specific pharmacotherapy. Central nodes (yellow) represent core pathophysiological processes common to multiple disorders: gut microbiota dysbiosis, intestinal barrier impairment, neuroinflammation, immune activation, BBB (BBB) dysfunction, and neurotransmitter dysregulation. Left nodes (blue) represent neurological and psychiatric conditions discussed in this review: PD, AD, MS, autism spectrum disorder (ASD), depression/anxiety, and stroke. Lines connecting central nodes to disease nodes indicate the strength of evidence and clinical relevance of each pathway to each disorder (solid lines: well-established; dashed lines: emerging or hypothesized). Right nodes (green) represent pharmacological intervention categories, grouped by their primary mechanism of action: microbiota-directed therapies (probiotics, FMT, antibiotics), metabolite-based therapies (SCFAs, BAs), immune-inflammatory modulators (TNF-α inhibitors, IL-6 blockers, NLRP3 inhibitors), neural pathway modulators (VNS, α7nAChR agonists), and barrier-targeting agents (tight junction modulators, BBB stabilizers). The diagram emphasizes that most interventions target specific pathophysiological mechanisms rather than individual diseases, supporting the rationale for developing mechanism-based therapies that may be effective across multiple conditions. For detailed descriptions of each intervention and its evidence base are are provided in Tables 1, 2.

Dysbiosis and systemic inflammatory events, which follow the post-stroke breakdown of the intestinal barrier, are the primary pathophysiology of the MGBA in stroke treatment, and worsen BBB damage and secondary brain injury (Ullah et al., 2023). One avenue the pharmacological research takes is to create neuroprotective drugs that stabilize the BBB. As an example, endogenous compounds N, N-dimethyltryptamine (MDMA) treatment according to animal research has shown that these compounds have a considerable effect on reducing BBB leakage, cerebral edema and neuroinflammation after stroke by acting on the state of σ-1 receptors (László et al., 2025). An additional strategy that can be seen to do well is the utilization of an alternative route of drug delivery instead of the BBB; one such route would be the nasal delivery of engineered nanoparticles capable of targeting specific brain region ischemic areas (Yin et al., 2026). These mechanism-based modalities exhibit a significant therapeutic efficacy in animal models and seem to sharply differ from the existing clinical stroke treatment, which mostly aims at reperfusion and does not have many useful neuroprotective agent. One of the largest challenges in translational medicine will be to translate such novel BBB protectants or drug delivery systems into clinical practice (Table 2 and Figure 3).

**Table 2:** Clinical pharmacological evidence targeting the gut–brain axis across neurological and psychiatric disorders.

Disease	Intervention	Pharmacological target	Study design	Main outcome	Pharmacological limitation	References
PD	Probiotics	Microbiota–SCFA axis	Small RCT	Improved constipation; modest motor benefit	Short duration; microbiota heterogeneity	Ding et al. (2023), Binder et al. (2025), and Jeong et al. (2025)
AD	Probiotics	Inflammation/metabolites	RCT	Improved MMSE scores	Small sample size	Beydoun et al. (2013)
MS	Probiotics	Immune modulation (Th17/Treg)	Pilot trial	Reduced inflammatory markers	No relapse endpoints	Qin et al. (2007) and Aisen et al. (2003)
Depression	Psychobiotics	HPA axis; inflammation	Meta-analysis	Reduced HAM-D scores	High heterogeneity	Zhang Z. et al. (2025) and He et al. (2026)
Anxiety disorders	Probiotics	Neurotransmitter precursors	Small RCT	Reduced anxiety scales	Short follow-up	Zhang Z. et al. (2025) and He et al. (2026)
Stroke	Microbiota modulation	Systemic inflammation	Observational	Correlation with outcome	Lack of causality	Hsiao et al. (2013), Zhang et al. (2026), and Kakinaka and Umeno (2020)

This table summarizes available clinical and translational evidence for pharmacological and microbiota-based interventions targeting the gut–brain axis. While preclinical findings are generally consistent, clinical outcomes remain heterogeneous, highlighting key translational challenges.

Post-stroke gut dysbiosis is increasingly recognized as a modulator of neurovascular injury. Experimental studies demonstrate that microbiota alterations following ischemic stroke amplify blood–brain barrier (BBB) disruption, promote peripheral immune cell infiltration, and exacerbate neuroinflammation, thereby worsening neurological outcomes (Benakis et al., 2016; Singh et al., 2016). Mechanistically, microbiota-derived signals influence endothelial tight junction integrity, systemic cytokine profiles, and microglial activation, positioning BBB stabilization as a central pharmacological objective (Braniste et al., 2014). One emerging pharmacological strategy involves activation of the sigma-1 receptor (Sig-1R), a ligand-operated chaperone protein localized at the endoplasmic reticulum–mitochondrial interface. Sig-1R activation modulates calcium homeostasis, attenuates oxidative stress, and suppresses pro-inflammatory signaling. Notably, N,N-dimethyltryptamine (DMT), an endogenous Sig-1R agonist, has been shown to reduce ischemia-induced neuronal injury and dampen inflammatory responses, suggesting a potential role in preserving neurovascular unit integrity, including BBB stability (Ruscher et al., 2011; Szabó et al., 2021). In parallel, advances in drug delivery science propose an orthogonal solution: bypassing BBB constraints. Intranasal administration enables direct nose-to-brain transport via olfactory and trigeminal neural pathways, facilitating CNS delivery while minimizing systemic exposure. Nanoparticle-based intranasal systems have demonstrated enhanced brain targeting, improved pharmacokinetic profiles, and increased drug accumulation in ischemic regions in preclinical stroke models (Lochhead and Thorne, 2012; Thorne et al., 2008).

### 4.7 Cross-disease pharmacological insights and translational barriers—summary

Across diverse neurological conditions—including PD, AD, autism spectrum disorder, and stroke—critically important and translationally limiting pharmacological themes recur:

First, indirect versus direct targeting. Many GBA-targeted interventions exert their effects indirectly via peripheral systems, relying on the modulation of circulating metabolites (e.g., SCFAs, BAs) or inflammatory mediators rather than direct CNS target engagement. This distinction has profound pharmacodynamic implications, as peripheral concentrations and mediator transport mechanisms (e.g., vagal signaling, BBB transcytosis) fundamentally determine central efficacy, in stark contrast to classic CNS drugs with optimized brain penetration (Cryan et al., 2019; Kim et al., 2023).

Second, pharmacokinetics and bioavailability as pervasive barriers. Pharmacokinetics and bioavailability remain pervasive barriers. Microbial metabolites such as SCFAs are rapidly metabolized and exhibit limited plasma persistence, and probiotic colonization is highly individualized, resulting in inconsistent exposure–response relationships (Zmora et al., 2018; Anand and Mande, 2022). Without rigorous PK/PD characterization (e.g., temporal concentration profiling, receptor occupancy or downstream biomarker engagement), mechanistic conclusions from intervention studies remain speculative.

Third, heterogeneity as the rule, not the exception. Inter-individual variability in host microbiota composition, immune status, and metabolic capacity contributes to differential therapeutic responses across AD, PD, and ASD cohorts, highlighting the need for microbiome phenotyping and stratified trial designs to identify “gut-driven” subpopulations with the highest likelihood of benefit (Ma et al., 2024; Franzosa et al., 2019; Ebadpour et al., 2025).

Fourth, the critical importance of timing. Animal models typically implement interventions at pre-symptomatic or early pathological stages, whereas clinical trials enroll participants with established or advanced disease, where irreversible neurodegeneration and entrenched inflammatory circuits limit pharmacological impact. This temporal discrepancy suggests that earlier, prodromal intervention may be necessary to reveal true disease-modifying potential (Jack et al., 2018; Salloway et al., 2022; Postuma and Berg, 2019).

Finally, the translation of GBA strategies will depend on integration of quantitative biomarkers (e.g., circulating metabolites, zonulin, neuroinflammatory markers) and trial designs that link mechanistic engagement to clinical outcomes. Only through this PK/PD-anchored, stratified, and temporally optimized framework can GBA-targeted pharmacotherapies move beyond proof-of-concept into reproducible clinical impact.

## 5 Challenges, limitations, and safety concerns in GBA-targeted pharmacotherapy

Despite the promising preclinical landscape and growing clinical interest in targeting the MGBA (GBA), several fundamental challenges and safety concerns must be addressed to facilitate successful clinical translation.

### 5.1 Mechanistic uncertainties and complexity

A primary limitation is the difficulty in establishing causality. Many human studies report associations between microbial taxa and disease states, but these do not prove a causal role in pathogenesis. Furthermore, the GBA is not a linear pathway but a complex, bidirectional network. Focusing on single microbial strains or metabolites may oversimplify this ecology, as the functional output of the microbiota (the “metabolome”) is an emergent property of the entire microbial community. Interventions that alter one component may have unintended consequences on others.

### 5.2 Pharmacokinetic and delivery barriers

As discussed in Section 3, many promising GBA-targeted agents face significant pharmacokinetic (PK) hurdles. SCFAs, for example, are rapidly absorbed and metabolized in the gut and liver, resulting in poor systemic bioavailability and making it difficult to achieve CNS-relevant concentrations. Probiotics face challenges of viability during gastrointestinal transit and highly variable colonization efficiency across individuals. For biologics (e.g., nanobodies), crossing the BBB (BBB) remains a formidable obstacle, requiring sophisticated engineering strategies such as receptor-mediated transcytosis. The field critically lacks robust pharmacokinetic/pharmacodynamic (PK/PD) studies that define the relationship between dose, exposure (e.g., plasma metabolite levels), and target engagement (e.g., changes in inflammatory markers).

### 5.3 Safety concerns

#### 5.3.1 Risks of pathway modulation

As with any potent biological pathway, targeted intervention carries risks. For instance, while activation of the WNT/β-catenin pathway has emerged as a promising strategy for stabilizing the BBB, this pathway plays a critical role in cell proliferation and stem cell maintenance across multiple tissues. Systemic or prolonged activation could theoretically promote aberrant cell growth or tumorigenesis. Recent comprehensive reviews have highlighted this dual role, emphasizing that therapeutic strategies must incorporate mechanisms for spatiotemporal control, such as CNS-targeted delivery or prodrug activation, to mitigate peripheral oncogenic risk (Jin et al., 2026; Faraji et al., 2025a).

#### 5.3.2 Chronic immunosuppression

Long-term inhibition of key inflammatory mediators like TNF-α or IL-6, while beneficial for neuroinflammation, carries well-documented risks of systemic immunosuppression, including increased susceptibility to infections and reactivation of latent pathogens.

#### 5.3.3 Unknowns of live biologics and FMT

The use of live organisms (probiotics) and complex microbial consortia (FMT) introduces unique safety challenges.

These include the potential for translocation of bacteria from the gut to the bloodstream in vulnerable individuals, the transfer of antibiotic resistance genes, and the long-term, unpredictable consequences of permanently altering a patient’s gut ecosystem.

### 5.4 Clinical and regulatory hurdles

#### 5.4.1 Patient heterogeneity

The high inter-individual variability of the human microbiome is a major source of inconsistent clinical trial results. This underscores the urgent need for stratified medicine approaches, where patients are selected based on baseline microbial or metabolic biomarkers (e.g., “leaky gut” phenotype, specific microbial signatures) that predict response.

#### 5.4.2 Placebo effect

In disorders with subjective endpoints, such as depression, anxiety, and IBS, the placebo response can be substantial, potentially masking true treatment effects or leading to false positives in poorly controlled trials.

#### 5.4.3 Regulatory pathways

Determining the appropriate regulatory pathway for novel GBA interventions is complex. Are live biotherapeutic products drugs, biologics, or medical foods? This ambiguity creates uncertainty for developers and can delay clinical adoption.

### 5.5 Future precision intervention pathways

Despite the challenges outlined above, several emerging strategies offer promising pathways toward clinical implementation of GBA-targeted pharmacotherapies within a precision medicine framework.

Patient stratification based on gut microbiome profiles. Inter-individual variability in microbiota composition is a major source of heterogeneous treatment responses. Stratifying patients based on baseline microbial signatures—such as the abundance of SCFA-producing taxa, gut permeability markers (e.g., serum zonulin), or specific pathogen presence—could identify “gut-driven” subpopulations most likely to benefit from microbiome-directed interventions (Ma et al., 2024; Ebadpour et al., 2025). Such stratification approaches are already being explored in proof-of-concept trials for PD and depression, where baseline microbiota profiles have been associated with differential responses to probiotics (Cryan et al., 2019; Barichella et al., 2016).

Metabolomics-guided probiotic selection. The functional output of the microbiome—the metabolome—may be a more reliable predictor of therapeutic response than taxonomic composition alone. Metabolomics-guided approaches aim to identify specific metabolite deficiencies (e.g., low butyrate, altered bile acid profiles) in individual patients and select probiotic strains or postbiotic formulations designed to correct these deficits (Cryan et al., 2019; Dalile et al., 2019). This moves beyond a “one-strain-fits-all” approach toward targeted metabolic reconstitution, with the potential for improved consistency and efficacy.

Machine learning-assisted target prediction and trial design. The complexity of the GBA—with its multiple interacting pathways, feedback loops, and inter-individual variability—makes traditional reductionist approaches insufficient for predicting treatment outcomes. Machine learning (ML) algorithms, trained on multi-omics datasets (metagenomics, metabolomics, proteomics, clinical data), offer the ability to identify non-linear patterns and predict individual responses to GBA-targeted interventions (Ma et al., 2024). ML models can also assist in identifying novel pharmacological targets by integrating large-scale genomic and functional data, accelerating drug discovery and repurposing efforts (Ebadpour et al., 2025).

Integrating these precision medicine tools into clinical trial design will be essential for moving GBA-targeted therapies beyond proof-of-concept. By identifying the right patient, with the right microbial and metabolic profile, and matching them to the right intervention, the field can begin to realize the promise of truly personalized GBA pharmacotherapy.

## 6 Conclusion

In conclusion, the MGBA has emerged as a critical nexus in the pathophysiology of a wide range of neurological and psychiatric disorders, offering a rich landscape for pharmacological innovation. This review has systematically mapped the key mechanistic pathways—from microbial metabolites to immune and neural signaling—and the corresponding therapeutic strategies under development. While preclinical evidence provides robust proof-of-concept, the translation to clinical practice is constrained by significant challenges, including the indirect nature of peripheral-to-central signaling, the lack of robust PK/PD data, the profound heterogeneity of the human microbiome, and critical safety considerations as discussed in Section 5. Addressing these limitations will require a concerted effort to develop stratified, biomarker-driven approaches and to design drugs with precise spatiotemporal activity.

The future of GBA-targeted pharmacotherapy lies not in a “one-size-fits-all” intervention, but in a precision medicine framework that integrates microbial, metabolic, and clinical data to deliver the right intervention to the right patient at the right time. By harnessing these principles, the next generation of GBA-directed therapies holds the potential to transform the treatment landscape for neurological and psychiatric disorders, moving from symptomatic management toward mechanism-based, disease-modifying interventions.
